# Kefir enhances stress resilience and mitigates PTSD-related behavioral and hematological changes in mice

**DOI:** 10.3389/fphys.2025.1682807

**Published:** 2025-11-14

**Authors:** Vitalii A. Balatskyi, Tetiana R. Dmytriv, Andrii Divnych, Volodymyr I. Lushchak

**Affiliations:** 1 Department of Biochemistry and Biotechnology, Vasyl Stefanyk Carpathian National University, Ivano-Frankivsk, Ukraine; 2 Research and Development University, Ivano-Frankivsk, Ukraine

**Keywords:** stress, anxiety, behavior, PTSD, open field test, foot shock, inflammation

## Abstract

Post-traumatic stress disorder (PTSD) is a complex psychiatric condition characterized by behavioral, cognitive, immunological, and neurochemical disturbances following traumatic experiences. Despite various therapeutic approaches, effective long-term treatments remain limited, highlighting the need for preventive strategies that enhance stress resilience. In this study, we evaluated the impact of long-term kefir consumption on behavioral, hematological, and biochemical parameters in a mouse model displaying some PTSD-like features, particularly fear- and anxiety-related behaviors induced by acute inescapable stress. Male C57BL/6J mice received kefir daily for 2 months before stress induction via electric foot shocks and continued supplementation for five additional months during recovery. Behavioral testing demonstrated that kefir-fed mice exhibited reduced anxiety-like behaviors, including increased exploration in the open field, elevated plus maze, and light/dark box tests. These mice also showed fewer freezing episodes in the aversive context test, indicating attenuated fear memory. Hematological analysis revealed a modest reduction in erythrocyte count and monocytes, alongside elevated paraoxonase (PON) activity, suggesting enhanced antioxidant defense and a shift toward anti-inflammatory immune responses. RT-qPCR analysis of the cerebral cortex showed increased steady-state transcript levels of genes involved in oxidative stress response and neuroprotection (TXNRD1, UGDH, HSPB8, GADD45B, PPARGC1A) and decreased levels of the pro-inflammatory cytokine gene IL6 transcript. These results indicate that long-term kefir intake mitigates stress-induced behavioral and physiological alterations, likely through modulation of immune and oxidative stress pathways. Taken together, our findings support the potential of kefir as a functional dietary intervention for promoting stress resilience and alleviating PTSD-like symptoms, possibly via mechanisms involving the gut-brain axis.

## 1 Introduction

Post-traumatic stress disorder (PTSD) is a multifaceted psychiatric condition that arises following exposure to traumatic events. It is characterized by intrusive memories, mood disturbances, cognitive impairments, hyperarousal, avoidance behavior, and a persistent sense of threat (Miao et al., 2018; Dmytriv et al., 2023; Pinna et al., 2023; Balatskyi et al., 2025). Individuals with PTSD are at increased risk of developing depression neurodegenerative diseases, substance use disorders, and various comorbidities. A similar pattern has recently been observed in the Ukrainian population affected by the ongoing Russian-Ukrainian war (Lushchak et al., 2023). Although preventive strategies are regarded as the most effective means of addressing PTSD, existing approaches lack sufficient evidence to support widespread clinical implementation (Bisson et al., 2021). Consequently, the development of novel, accessible interventions to promote stress resilience remains a research priority.

Dietary interventions targeting mental health, particularly PTSD, are still underexplored, despite growing recognition of the gut-brain axis as a central regulator of stress responses and emotional wellbeing (Yin et al., 2014; Kearney et al., 2022; Rook, 2024). Functional foods, particularly those rich in probiotics and bioactive compounds, have demonstrated the potential to modulate brain function through microbial metabolites and neuroactive compounds (Gomez-Pinilla and Gomez, 2011; Gradus et al., 2017). Fermented dairy products (FDPs) are of particular interest due to their ability to influence the composition and function of the gut microbiota. It is also known that kefir consumption by mice had a positive effect on inflammation modulation by reducing the level of pro-inflammatory cytokines and increasing the level of anti-inflammatory cytokines, as well as modulating oxidative stress (Gogineni, 2013; Albuquerque Pereira et al., 2024; Mariana et al., 2024). Experimental studies have shown that peptides derived from fermented milk can reduce anxiety-like behavior and mitigate brain damage in stress-exposed mice (Joung et al., 2021; 2023). Moreover, human studies report associations between FDP consumption and lower anxiety levels (Sousa et al., 2022). These benefits are largely attributed to modulation of the gut-brain axis–a – bidirectional communication system linking the gastrointestinal tract and central nervous system via immune, neural, and endocrine pathways (Dmytriv et al., 2024a; Dmytriv et al., 2024b; Loh et al., 2024). However, the long-term effects of FDPs on stress-related behavior and their underlying molecular mechanisms remain poorly characterized.

While many studies have focused on the microbial or metabolic mechanisms of fermented products, the initial step in our research was to determine whether specific kefir as a whole could induce measurable behavioral and physiological benefits in a murine PTSD-like model. This approach allowed us to assess the integrated biological impact of kefir before dissecting the contributions of its individual microbial or biochemical components. Subsequent studies will focus on identifying the specific active factors and pathways underlying these effects.

In this study, we investigated whether prolonged kefir consumption could attenuate stress-induced behavioral, hematological, and molecular alterations in a mouse model of PTSD. Our findings provide compelling evidence that kefir may serve as a functional dietary intervention to enhance stress resilience and mitigate the symptoms of PTSD.

## 2 Materials and methods

### 2.1 Experimental design

Male C57BL/6J mice aged 8–11 months were housed under controlled laboratory conditions with a 12-h light/dark cycle (6 a.m.–6 p.m.), ambient temperature of 22 °C ± 2 °C, and relative humidity of 50%–60%. Mice were randomly assigned to control and experimental groups (five to seven animals per group). Control animals received standard rodent chow (21.8% protein, 4.8% fat, 69.1% carbohydrates, 3.9% fiber), while the experimental group received unlimited access to chow and kefir in separate dishes. Food, water, and kefir were provided *ad libitum*.

Kefir (Molokiya, Ukraine) was prepared from normalized cow’s milk with kefir starter culture. Nutritional values per 100 g: carbohydrates – 3.9 g (including 3.9 g sugars), proteins – 2.9 g, fats – 2.5 g (1.58 g saturated), salt – 0.05 g; energy value – 209 kJ (50 kcal).

**FIGURE 1:**
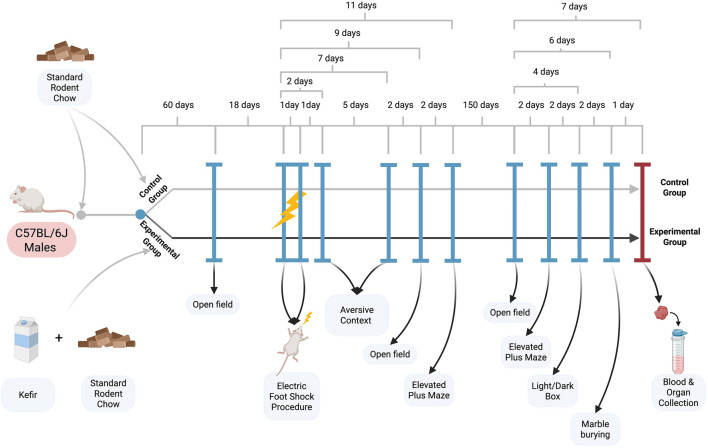
Experimental scheme.

After 2 months of kefir consumption, mice underwent baseline behavioral testing (open field test) before stress induction. Mice then received two sessions of electric foot shocks over 2 days to induce PTSD-like symptoms and continued kefir intake for five additional months. Behavioral tests were conducted at several time points (see Figure 1): the aversive context test (days 2 and 7), open field test (day 9), and elevated plus maze (day 11). Long-term behavioral effects were assessed 5 months later using the open field, elevated plus maze, light/dark box, and marble burying tests.

All procedures were approved by the Animal Experiments Committee of Vasyl Stefanyk Precarpathian National University and conducted under Directive 2010/63/EU on the protection of animals used for scientific purposes.

### 2.2 Stress induction procedure and aversive context test

Stress was induced using a metal-grid floor shock chamber connected to a stimulus generator. This acute footshock paradigm is widely used to model trauma-induced fear and anxiety in rodents and is often referred to as a PTSD-like or contextual fear model (Martinho et al., 2021). It does not reproduce all parameters of human PTSD, but allows the assessment of persistent fear memory and stress reactivity. Each mouse was placed in the chamber for 7 min: 2 min of acclimatization followed by 15 electric shocks (0.8 mA, 10 s duration, 10 s intervals) over 5 min, repeated on two consecutive days (Martinho et al., 2022). On days 2 and 7, mice were re-exposed to the same chamber without shocks for the aversive context test. Freezing behavior, defined as immobility except for breathing for ≥3 s, was scored from video recordings (Martinho et al., 2021).

### 2.3 Open field test

The open field test is commonly used to measure locomotor and anxiety-like behavior in mice (Seibenhener and Wooten, 2015). In this work behavioral activity was assessed in a 40 × 40 cm polyvinyl chloride chamber divided into 16 squares (10 × 10 cm). Locomotion and anxiety-related behaviors were recorded and analyzed using ToxTrac software (v2.98) from 10-min video recordings (Rodriguez et al., 2018; https://sourceforge.net/projects/toxtrac/). Outcomes included average movement speed, time spent in the central squares (inner zone), and number of fecal boli.

### 2.4 Elevated plus maze test

Anxiety-related behavior was evaluated using a standard elevated plus maze (EPM) with two open and two closed arms intersecting at a central platform. Mice were allowed to explore the maze for 10 min. The time spent in open and closed arms was recorded (Walf and Frye, 2007).

### 2.5 Light/dark box test

Mice were placed in a divided glass box (30 × 30 × 40 cm per compartment) with one dark and one illuminated zone. After being placed in the dark zone and closing the lid, mice were observed for 10 min. The number of entries into the light zone, time spent there, and latency to first entry were recorded (Bourin and Hascoët, 2003; Crawley and Goodwin, 1980).

### 2.6 Marble burying test

Each cage was filled with 5 cm of wood shavings, and 20 marbles were arranged in four rows. Mice were placed in the cage for 30 min, and the number of marbles buried ≥75% was counted (Angoa-Pérez et al., 2013; Sampson, 2024).

### 2.7 Hematological parameters

Blood was collected after a 12-h fast via retro-orbital puncture under CO_2_ anesthesia. Half the sample was centrifuged (1500 g, 15 min, 4 °C) for plasma; the remainder was used for hematological analyses.

Hemoglobin was measured using Drabkin’s reagent (Genesis LLC, Ukraine) at 540 nm. Hematocrit was assessed using microcapillary tubes centrifuged at 2000 *g* for 20 min. Erythrocyte and leukocyte counts were performed using Goryaev’s chamber after dilution with 3% NaCl or 5% acetic acid + methylene blue, respectively. Leukocyte differentials were determined from blood smears stained by Romanowski or May-Grunewald-Giemsa methods, counting 200 cells per animal at 1000× magnification. The cells were classified according to standard protocols (O’Connell et al., 2015), and the percentages of various leukocyte types were determined.

### 2.8 Assays of activities of paraoxonase, myeloperoxidase, and levels of total protein and glucose in blood plasma

Plasma paraoxonase (PON) activity was measured spectrophotometrically at 405 nm using 4-nitrophenyl acetate as a substrate. The reaction mixture consisted of 50 mM potassium phosphate buffer (pH 7.0), 1 mM CaCl_2_, and 3.2 mM 4-nitrophenylacetate. The extinction coefficient of p-nitrophenol 14,000 M^-1^ cm^-1^, was used to calculate the PON activity (Vatashchuk et al., 2023). Myeloperoxidase (MPO) activity was measured as H_2_O_2_-dependent oxidation of 3,3′,5,5′-tetramethylbenzidine (TMB), and the absorbance was measured at 450 nm using a Multiskan MCC/340 microplate reader (Yadav et al., 2014). Plasma glucose and total protein levels were assessed using standard kits and the Bradford assay (Bradford, 1976).

### 2.9 Polymerase chain reaction (RT-qPCR)

Total RNA isolation, RNA quantification, and real-time quantitative polymerase chain reaction (RT-qPCR) were performed as previously described (Demianchuk et al., 2024). The AriaMx system (Agilent Technologies, Inc.) was used for RT-qPCR. Relative fold change in messenger RNA (mRNA) levels was calculated using the 2–ΔΔCq method (Livak and Schmittgen, 2001), using Cq values for the expression of the RPL27 gene (encoding ribosomal protein L27) as a reference.

Total RNA was extracted from cerebral cortex samples as previously described (Demianchuk et al., 2024). RT-qPCR was performed using the AriaMx system (Agilent Technologies). Relative transcript levels of genes of interest gene expression were calculated using the 2–ΔΔCq method (Livak and Schmittgen, 2001), normalized to *RPL27* as a reference gene. Oligonucleotide sequences (see Table 1) were received from Metabion International AG (Steinkirchen, Germany).

**TABLE 1:** Oligonucleotide sequences used in the study to analyze the levels of mRNA by quantitative real-time polymerase chain reaction.

Gene	Forward primer (5′→ 3′)	Reverse primer (5′→ 3′)
TXNRD1	GACTGGCAGCAGCTAAGGA	GAGCTTGTCCGAGCAAAGC
GSTM3	TATGGACACCCGCATACAGC	GCTTCATTTTCTCAGGGATGGC
UGDH	TGCATGGAATTCTCCAACCT	AGATCGGCTTCTCTGATGGC
HSPB8	CGTGGAAGTTTCAGGCAAACA	CACTTCTGCAGGGAGCTGTAT
GADD45B	TGAATGTGGACCCCGACAG	AGCAGAACGACTGGATCAGG
SQSTM1	CTACCGCGATGAGGATGGG	CACAGATCACATTGGGGTGC
BECN1	CCAGGAACTCACAGCTCCAT	ACCATCCTGGCGAGTTTCAA
CCL2	CAGCCAGATGCAGTTAACGC	TTCCTTCTTGGGGTCAGCAC
IL1B	TGAAGAAGAGCCCATCCTCTG	TCATATGGGTCCGACAGCAC
IL-6	CCGGAGAGGAGACTTCACAG	CCACGATTTCCCAGAGAACATG
SGK1	GAACCACGGGCTCGATTCTA	CAGATACTCAGGCGTGCCA
S100A10	GGCGACAAAGACCACTTGAC	GAAGCCCACTTTGCCATCTC
SHANK1	GCAGACCATCAGTGCAAGTG	AGCCCCGATAGATTTCTGCC
STK11	CCTACTCCGAGGGATGTTGG	AGCTGTGCTGCCTAATCTGT
NR3C1 (GR)	ACTGCTTCTCTCCTCAGTTCC	TCTGACTGGAGTTTCCTTCCC
FKBP5	AGTCAATCCTCAGAACAGGGC	CTTTGCTGGCTTCCTCCTTTG
RICTOR	CGCTCGTGGGCAGGTATTAT	GGATCTACACTGAGCAGGGC
4EBP	CTCCTGGAGGCACCAAACT	CTTGATCAGTTCCGTGGGGA
PPARGC1A	CTCTGGAACTGCAGGCCTAA	GCCTTGGGTACCAGAACACT
BNIP3	ACAGCACTCTGTCTGAGGAAG	TGCTGAGAGTAGCTGTGCG
CD36	GGCTAAATGAGACTGGGACCA	TCTCTACCATGCCAAGGAGC
RPL27	TCAGGGACCCAGCTTTGAAG	TTCCCTGTCTTGTATCGCTCC

The prefrontal cortex was selected for molecular analysis due to its key involvement in emotional regulation, cognitive control, and extinction of fear responses, which are central to PTSD pathology. This region also provides sufficient tissue quantity for reproducible RNA isolation and reliable RT-qPCR measurements.

### 2.10 Statistical analysis

Data are presented as mean ± SEM. Statistical analyses were conducted using GraphPad Prism v10.0.0 (GraphPad Software, Boston, MA, USA). Differences between groups were assessed using unpaired Student’s t-test, Holm-Sidak test. Linear mixed effects model approach implemented in GraphPad Prism was applied to evaluate time-dependent influence of the treatment. Multiple comparisons were conducted using t-test followed by p-value adjustment by Benjamini-Krieger-Yekutieli procedure.

Sample size was evaluated using Robin Ristl’s sample size calculator (Medical University of Vienna, https://homepage.univie.ac.at/robin.ristl/samplesize.php?test=anova), for two groups with unequal sample sizes, with mean difference 1.5, standard deviation of 0.6, significance level 0.05, and power 0.8. This calculation gave us a sample size of four and six individuals.

## 3 Results

### 3.1 Body mass and Fur condition of mice

**FIGURE 2:**
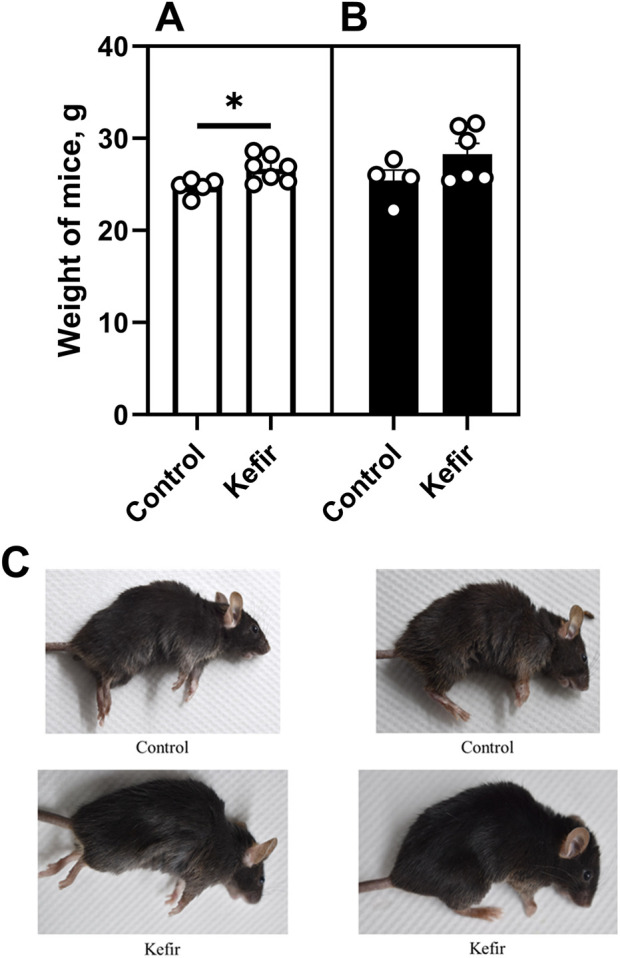
Body mass of mice **(A)** before the foot shock procedure, **(B)**, at the end of the experiment, and **(C)** fur conditions of animals from the control and kefir-consuming group under anesthesia. Data are presented as mean ± SEM, *n =* 4-6. *Significantly different from the control group (P < 0.05) according to unpaired Student’s t-test.

Before the first open field test induction (conducted prior to the foot shock procedure), kefir-fed mice had significantly higher body mass–approximately 8% (p = 0.01) greater–compared to control animals (Figure 2A). However, by the end of the experiment, body mass differences were no longer statistically significant (Figure 2B). Kefir-treated mice also exhibited a noticeably improved fur conditions, including increased physical activity, livelier behavior, and shinier fur compared to controls (Figure 2C).

### 3.2 Behavioral tests

**FIGURE 3:**
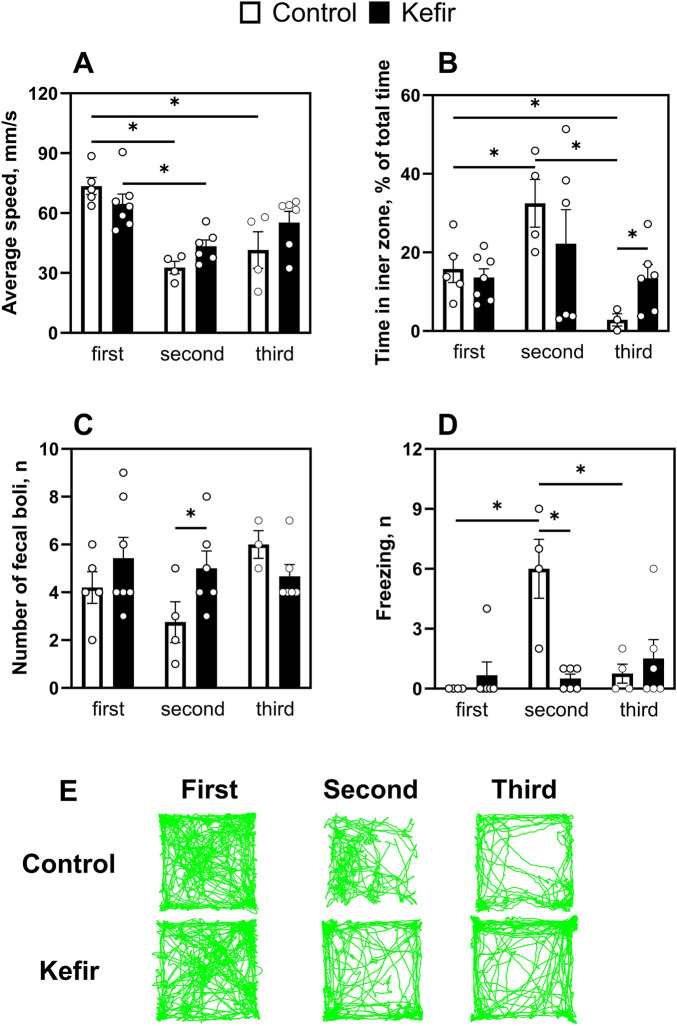
Comparison of results of the first (before foot shock), second (1 week after foot shock), and third (5 months after foot shock) open field test trails. **(A)** An average speed of mice, **(B)** time spent in the central squares of the open field, **(C)** number of fecal boli, **(D)** number of freezings of mice, **(E)** representative trails of mouse movement. Data are presented as mean ± SEM, *n =* 3-7. *Significant difference (P < 0.05) between groups according to the mixed effect model approach, followed by pairwise comparisons with Benjamini-Krieger-Yekutieli adjustment of *p*-values for multiple testing.

The open field test was conducted three times: before stress exposure, 1 week post-stress, and 5 months after stress induction (Figure 1). Control mice displayed lower locomotor activity after stress, with average speed decreased by 56% (p < 0.001) in the second trial and 44% (p < 0.001) in the third compared to the first. Kefir-treated mice also exhibited a 33% (p = 0.005) lower speed in the second test compared to pre-stress levels, but their behavior remained more consistent over time (Figure 3A).

In terms of anxiety-like behavior, control mice spent twice as much time in the inner zone during the second test compared to the first (p = 0.03). However, their time in the center markedly decreased in the third trial - to 82% (p = 0.044) and 91.3% (p = 0.04) of the values observed in the first and second tests, respectively. In contrast, kefir-fed mice maintained stable exploration of the central area across all trials and spent nearly five times more time (p = 0.03) in the inner zone than controls during the third test (Figure 3B), suggesting reduced anxiety. The number of fecal boli did not differ significantly, except during the second trial, when the kefir group produced nearly twice as many (p = 0.04) (Figure 3C). Freezing behavior peaked in control animals during the second trial, while kefir-fed mice exhibited consistently lower freezing, including significantly fewer episodes than controls during the second test (p < 0.001) (Figure 3D).

**FIGURE 4:**
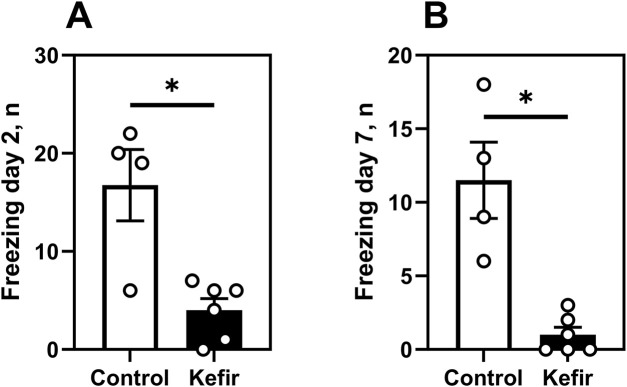
Aversive context test. **(A)** number of freezing events of mice in the aversive context on day 2, **(B)** number of freezing events of mice in the aversive context on day 7. Data are presented as mean ± SEM, *n =* 4-6. *Significantly different from the control group (P < 0.05) according to unpaired Student’s t-test.

In the aversive context test, kefir-fed mice showed markedly lower fear responses, with 76% fewer freezing episodes (p = 0.03) on day 2% and 91% fewer on day 7 (p = 0.02) compared to control mice (Figures 4A,B), indicating sustained attenuation of fear-related responses.

**FIGURE 5:**
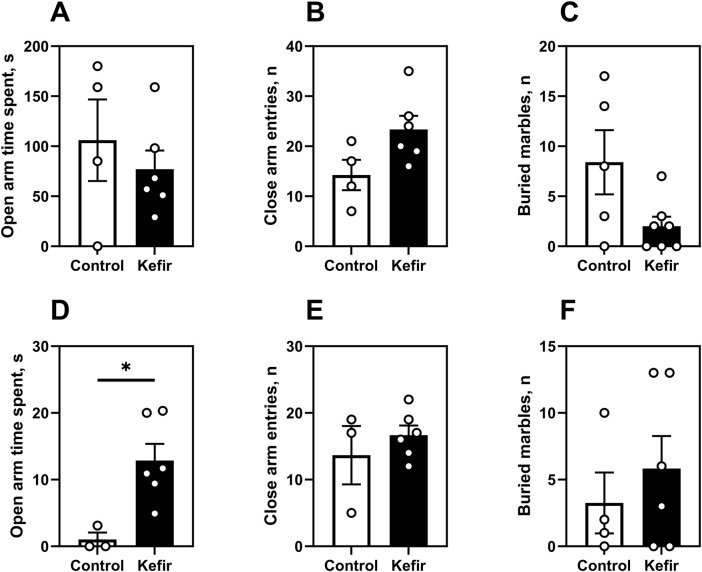
Elevated plus maze and marble burying tests. This figure shows data from two trials of the elevated plus maze test. The first trial was conducted 1 month after the foot shock **(A–C)**, and the second 5 months after the foot shock **(D,E and F)**. First trial. **(A)** Time spent in the open arms of the elevated plus maze, **(B)** number of closed arm entries of the elevated plus maze, **(C)** number of buried marbles in the marbles burying test. Second trial. **(D)** time spent in the open arms of the elevated plus maze, **(E)** number of entries into the closed arms of the elevated plus maze, **(F)** number of balls buried in the ball burial test. Data are presented as mean ± SEM, *n =* 3-7. *Significantly different from the control group (P < 0.05) according to unpaired Student’s t-test.

In the first round of elevated plus maze (EPM) and marble burying tests (1 week after foot shock), no significant differences were observed between groups (Figures 5A–C). However, in the second round (5 months post-stress), kefir-fed mice spent 12 times more time (p = 0.004) in the open arms of the EPM compared to controls (Figure 5D), indicating reduced anxiety. The number of entries into closed arms did not differ (Figure 5E), nor did marble burying behavior (Figure 5F).

**FIGURE 6:**
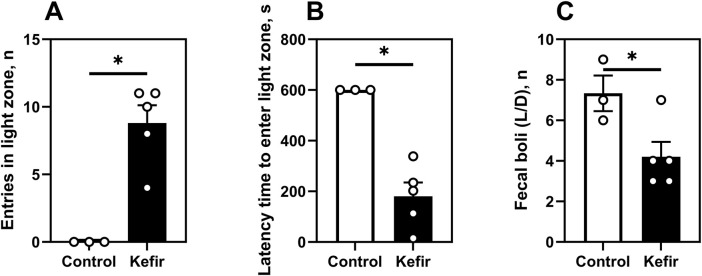
Light/dark box test (5 months after foot shock). **(A)** number of entries in the light zone of the light/dark box, **(B)** latency time (time spent by mice to enter the light zone for the first time), **(C)** number of mice defecating in the light/dark box. Data are presented as mean ± SEM, *n =* 4-6. *Significantly different from the control group (P < 0.05) according to unpaired Student’s t-test.

In the light/dark box test (5 months post-stress), kefir-fed mice made significantly more entries (p = 0.003) into the light zone and exhibited shorter latency to enter it (p = 0.002), compared to controls (Figures 6A,B). These mice also defecated 43% less often during the test (p = 0.04) (Figure 6C), further supporting an anxiolytic effect of kefir.

### 3.3 Hematological parameters

Among hematological parameters, only red blood cell count was significantly lower (by 17%) in kefir-consuming mice compared to controls (p = 0.001) (Table 2). No significant differences were found in hemoglobin concentration, hematocrit, or total leukocyte count between the groups.

**TABLE 2:** Hematological parameters in peripheral blood from mice of the control and kefir-fed groups.

Parameter	Control	Kefir
Hemoglobin, g/L	146 ± 3	154 ± 3
Hematocrit, %	47.4 ± 0.51	48.4 ± 1.49
Erythrocyte count, 10^6^/mL	7.38 ± 0.18	6.12 ± 0.17*
Total leukocyte count, 10^3^/mL	5.20 ± 0.74	4.73 ± 0.44

Data are presented as mean ± SEM, *n*= 4-6. *Significantly different from the control group (P < 0.05) according to unpaired Student’s t-test.

Differential leukocyte analysis showed a 50% lower monocyte percentage (p = 0.03) and a 27% higher juvenile leukocyte form (p = 0.01) in the kefir-fed group (Table 3). Percentages of other leukocyte subtypes (lymphocytes, segmented and banded neutrophils) did not differ between groups.

**TABLE 3:** Leukocyte formula of peripheral blood from mice of the control and kefir-fed groups.

Leukocyte type, %	Control	Kefir
Juvenile forms	4.88 ± 0.24	6.20 ± 0.25*
Banded neutrophils	3.50 ± 1.0	2.90 ± 0.2
Segmented neutrophils	9.25 ± 2.39	11.7 ± 1.17
Basophils	NF	NF
Eosinophils	NF	NF
Lymphocytes	81.8 ± 3.1	79.5 ± 1.1
Monocytes	1.0 ± 0	0.50 ± 0.16*

Data are presented as mean ± SEM, *n*= 4-6. *Significantly different from the control group (P < 0.05) according to unpaired Student’s t-test. NF–not found.

### 3.4 Biochemical and metabolic parameters of blood plasma

**FIGURE 7:**
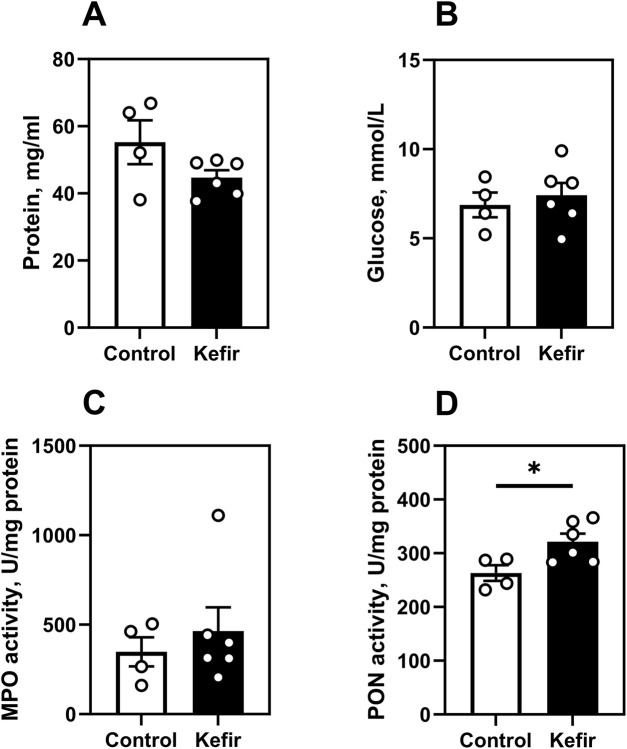
Metabolic and biochemical blood parameters. **(A)** total protein, **(B)** glucose level, **(C)** myeloperoxidase activity, **(D)** paraoxonase activity. Data are presented as mean ± SEM, *n =* 4-6. *Significantly different from the control group (P < 0.05) according to unpaired Student’s t-test.

No significant intergroup differences were detected in blood glucose, total plasma protein, or myeloperoxidase (MPO) activity (Figures 7A–C). However, paraoxonase (PON) activity was 22% higher in the kefir group (p = 0.03), indicating enhanced antioxidant capacity (Figure 7D).

### 3.5 mRNA levels in the mouse cerebral cortex

RT-qPCR analysis of cerebral cortex tissue revealed significant transcriptional differences between control and kefir-fed groups in transcript levels of genes involved in oxidative stress response, inflammation, neuroplasticity, autophagy, and energy metabolism (Table 4). Among antioxidant defense genes, *TXNRD1* and *UGDH* were upregulated by 34% (p = 0.049) and 40% (p < 0.001), respectively. Expression of *GSTM3* remained unchanged.

**TABLE 4:** mRNA transcripts in the cerebral cortex of mice. Data are presented as mean ± SEM, n = 3-5.

Role	Gene	Product	Control	Kefir
PTSD markers	SGK1	Serum/glucocorticoid regulated kinase 1	1.09 ± 0.26	0.96 ± 0.15
	NR3C1 (GR)	Nuclear receptor subfamily 3 group C member 1	1.02 ± 0.11	1.05 ± 0.10
	FKBP5	FK506 binding protein 5	0.84 ± 0.18	1.31 ± 0.10
	S100A10	S100 calcium binding protein A10	1.03 ± 0.14	0.55 ± 0.04*
	SHANK1	SH3 and multiple ankyrin repeat domains 1	1.18 ± 0.08	1.68 ± 0.09*
Protein synthesis and folding	HSPB8	Heat shock protein beta-8	0.88 ± 0.09	1.27 ± 0.05*
	4EBP	Eukaryotic translation initiation factor 4E-binding protein 1	0.86 ± 0.04	1.85 ± 0.20
Autophagy and TFEB targets	BECN1	Beclin-1	1.00 ± 0.02	1.32 ± 0.03*
	SQSTM1	Sequestosome-1	1.04 ± 0.15	1.21 ± 0.10
	BNIP3	BCL2 interacting protein 3	1.00 ± 0.02	1.32 ± 0.03
FOXO targets	RICTOR	Rapamycin-insensitive companion of mammalian target of rapamycin	1.03 ± 0.13	1.89 ± 0.07
	STK11	Serine/threonine kinase 11	1.03 ± 0.13	1.02 ± 0.05
	PPARGC1A	Peroxisome proliferator-activated receptor gamma coactivator 1-alpha	0.93 ± 0.13	1.41 ± 0.13*
	GADD45B	Growth arrest and DNA-damage-inducible beta	1.01 ± 0.06	1.50 ± 0.10*
Antioxidant response and xenobiotic detoxication	TXNRD1	Thioredoxin reductase 1	1.01 ± 0.08	1.35 ± 0.11*
	GSTM3	Glutathione S-transferase M3	1.01 ± 0.07	1.00 ± 0.03
	UGDH	UDP-glucose 6 -dehydrogenase	1.00 ± 0.03	1.40 ± 0.04*
Markers of inflammation and NF-κB targets	CCL2	C-C motif chemokine ligand 2	0.71 ± 0.01	1.03 ± 0.19
	IL1B	Interleukin 1 beta	1.09 ± 0.25	0.69 ± 0.01
	IL-6	Interleukin 6	0.87 ± 0.13	0.41 ± 0.06*
	CD36	Fatty acid translocase	1.29 ± 0.45	1.08 ± 0.29

*Significantly different from the control group (P < 0.05) according to unpaired Student’s t-test.

Pro-inflammatory *IL6* expression was lower by 53% (p = 0.046), while *IL1B* showed a non-significant 37% decrease (p = 0.2) (Table 4). *CCL2* levels were 45% higher (p = 0.18), possibly reflecting a compensatory immune mechanism. No significant differences were observed in *SGK1*, *NR3C1*, or *FKBP5* (glucocorticoid signaling pathway).

Neuroplasticity-related genes showed notable differences: *SHANK1* and *GADD45B* were upregulated by 43% (p = 0.006) and 50% (p = 0.004), respectively, while *S100A10* was downregulated (p = 0.04) by 47%. Among autophagy-related genes, *BECN1* was higher by 32% (p < 0.001), while *SQSTM1* and *BNIP3* levels remained unchanged (Table 4).

For protein homeostasis, *HSPB8* was 44% higher (p = 0.03). Genes related to mTOR signaling and metabolic regulation also responded to kefir intake: *RICTOR* was 84% higher (p = 0.002), *EIF4EBP1* nearly doubled, and *PPARGC1A* was elevated by 52% (p = 0.04). No significant differences were observed in *STK11* or *CD36* (Table 4).

## 4 Discussion

Fermented dairy products, particularly kefir, are increasingly recognized for their multifunctional health benefits, including immune modulation, antioxidant enhancement, and potential neuroprotective effects (Moineau and Goulet, 1991; Mitsuoka, 2014; Silva et al., 2023). These effects are largely attributed to kefir’s ability to modulate the gut microbiota and influence gut-brain axis communication (Perdigón et al., 2002; Kim et al., 2016; Kumar et al., 2021; Chakrabarti et al., 2022). The present study demonstrates that long-term kefir consumption exerts beneficial effects on behavior, physiology, and brain gene expression in a mouse model of post-traumatic stress disorder (PTSD).

In our work, kefir-fed mice exhibited improved fur conditions, and their body weight was also 8% higher compared to the control group. Therefore, we believe that this may be due to both the addition of kefir to their diet and the fact that the experimental group received more calories, which could have led to an improvement in their appearance. The mice also showed enhanced stress resilience, as indicated by stable exploratory activity and attenuated anxiety-like behavior across multiple behavioral tests, including the open field, elevated plus maze, light/dark box, and aversive context tests. In contrast, control mice displayed significantly lower locomotion and higher signs of anxiety, such as reduced central exploration and elevated freezing responses. These behavioral improvements align with previous reports showing that kefir modulates central neurotransmission through gut microbiota-derived metabolites, particularly those affecting GABAergic and serotonergic systems (Van De Wouw et al., 2020; Icer et al., 2024). Kefir peptides have also been shown to activate BDNF/TrkB signaling and reduce stress-induced hyperthermia, further supporting its role in stress resilience (Chen et al., 2021; Balasubramanian et al., 2024). An interesting effect of kefir on the number of defecations by mice in an open field test was also observed, since only in the second test (after stress) did the number of defecations increase in mice that consumed kefir. Although it was previously noted that kefir did not affect intestinal motility (Van De Wouw et al., 2020). This can be explained by the combination of kefir consumption and stress. For example, kefir lowers the pH in the large intestine due to the presence of short-chain fatty acids (butyric, propionic, and acetic acids), which, in turn, increases the secretion of corticosteroids in mice due to stress, and which can affect intestinal motility (Unsal and Balkay, 2012). Together, this can increase peristalsis, which is why mice had a higher level of defecation compared to the control group. However, after a certain period of time, the number of defecations normalized due to adaptation.

However, we did not observe any differences in the marble ball digging test, which is used to determine the presence of compulsive behavior. These results may be related to the involvement of different neural circuits (Shin and Liberzon, 2010): anxiety and fear are mainly associated with hyperactivation of the amygdala, while obsessive-compulsive disorder has a different localization in brain circuits, particularly the cortico-striato-thalamo-cortical loops (Li and Mody, 2016), and kefir may not have an effect on these regulatory areas of the brain. It may also be due to insufficient sensitivity of the test or an insufficient sample size for this test.

At the systemic level, kefir supplementation resulted in a modest reduction in erythrocyte counts without affecting hemoglobin or hematocrit, indicating preserved oxygen transport. Lower levels in monocytes and higher in juvenile leukocytes suggest a shift toward a less inflammatory immune profile, consistent with kefir’s known immunomodulatory effects (Karaffová et al., 2021; Ben Taheur et al., 2022).

Biochemical analyses showed that kefir did not affect either plasma glucose and protein levels, nor MPO activity, suggesting no acute systemic inflammatory responses. However, higher paraoxonase (PON1) activity in kefir-treated mice points to enhanced antioxidant defense and lipid metabolism, which may contribute to both cardiovascular and neuroprotective effects (Jakubowski, 2024).

In the mouse cerebral cortex, kefir consumption affected the expression of several genes related to oxidative stress response, inflammation, synaptic plasticity, and cellular metabolism. We observed an increase in mRNA levels of *TXNRD1* and *UGDH*, both associated with the Nrf2 pathway, which regulates cellular antioxidant defense and metabolic adaptation (Tonelli et al., 2018; Wu et al., 2012). These transcriptional changes may indicate activation of protective molecular responses rather than direct antioxidant effects.

A moderate reduction in *IL6* expression could reflect attenuation of neuroinflammatory signaling, possibly mediated by Nrf2–NF-κB crosstalk (Saha et al., 2020). In parallel, changes in *BECN1*, *HSPB8*, and *GADD45B* transcripts suggest a potential modulation of autophagy and stress adaptation pathways, although the functional implications remain to be clarified.

Genes involved in neuronal plasticity and energy metabolism, such as *SHANK1*, *RICTOR*, *EIF4EBP1*, and *PPARGC1A*, also showed altered transcripts. Since these genes participate in mTORC2 and mitochondrial biogenesis pathways, modulation of their expression might reflect compensatory cellular responses to the stress (Costa-Mattioli and Monteggia, 2013; Huang and Fingar, 2014).

Overall, the observed transcriptional changes suggest that kefir may influence molecular networks linked to antioxidant defense, inflammation, and synaptic regulation. However, as mRNA levels do not necessarily reflect protein abundance or enzymatic activity, these findings should be interpreted cautiously and verified in future studies at the protein or functional level.

The molecular analyses were limited to the prefrontal cortex because of its central role in cognitive control and emotional regulation in PTSD. Other regions, such as the hippocampus and amygdala, were not examined in the present series due to limited tissue availability. Nonetheless, future studies will include these regions to provide a more comprehensive neuroanatomical understanding of kefir’s effects.

Taken together, our findings indicate that long-term kefir consumption exerts broad protective effects in a PTSD-like mouse model by improving behavioral responses, modulating immune and oxidative stress markers, and enhancing the expression of genes involved in neuroplasticity and metabolic regulation. These results support the potential of kefir consumption as a functional dietary strategy for enhancing stress resilience and mitigating trauma-related disorders, likely through mechanisms involving the gut-brain axis. However, these findings also demonstrate the need for further research into optimized dosing, strain selection, and combinatorial therapies.

Further research on this topic should determine whether there is a direct correlation between kefir modulation of the microbiome and the results we obtained. 16S rRNA sequencing should be performed to identify the diversity of the microbiota, verify the expression of tight junction proteins occludin and ZO-1 in colon tissue, and study intestinal metabolites such as short-chain fatty acids and the tryptophan metabolite 5-hydroxytryptamine. In addition, our study determined the overall effect of kefir as a dietary supplement. Therefore, future research will aim to identify specific strains and metabolites responsible for the observed effects, which is critical for translational applications.

## 5 Conclusion and perspectives

This study demonstrates that long-term kefir consumption significantly attenuates stress-induced behavioral alterations and favorably modulates physiological and molecular parameters in mice exposed to traumatic stress. Kefir-treated mice exhibited reduced anxiety-like behavior across multiple validated tests, including the open field, elevated plus maze, light/dark box, and aversive context paradigms. In addition to behavioral improvements, kefir intake led to beneficial changes in hematological and biochemical markers, including increased paraoxonase activity and reduced monocyte levels, indicating enhanced antioxidant defense and lower systemic inflammation. At the molecular level, in the cerebral cortex, kefir modulated the expression of key genes associated with oxidative stress resistance, neuroprotection, synaptic plasticity, autophagy, and metabolic regulation. These findings suggest that kefir promotes stress resilience through the activation of adaptive pathways in both the immune and nervous systems, potentially mediated by the gut-brain axis. Collectively, our data support the potential of kefir as a functional dietary intervention to prevent or mitigate PTSD-related symptoms. While the present results are preliminary and limited to an animal model, they raise the possibility that kefir generally, and particular kefir Molokia, could contribute to nutrition-based strategies aimed at supporting human mental health and resilience.

This study highlights kefir’s potential as a functional dietary intervention for enhancing stress resilience. Future research should focus on identifying the specific microbial strains and metabolites responsible for its effects, as well as determining optimal dosage and duration. Kefir may contribute to improved stress resilience in mice, which warrants further exploration in the context of mental health. Additionally, combining kefir with other nutritional or therapeutic strategies may further enhance its benefits. Overall, kefir represents a promising, accessible tool for supporting mental health through gut-brain axis modulation.

## Data Availability

The raw data supporting the conclusions of this article will be made available by the authors, without undue reservation.
